# Supplementary material for: Reduction in Acute Bee Paralysis Virus Infection and Mortality in Honey Bees (Apis mellifera) by RNA Interference Technology
Source: Insects. 2025 Apr 25;16(5):453. doi: 10.3390/insects16050453 (PMC12112043; doi:10.3390/insects16050453)
Supplement: Supplementary file 1 [file insects-16-00453-s001.zip › insects-3389991-supplementary.pdf]

Supplementary material

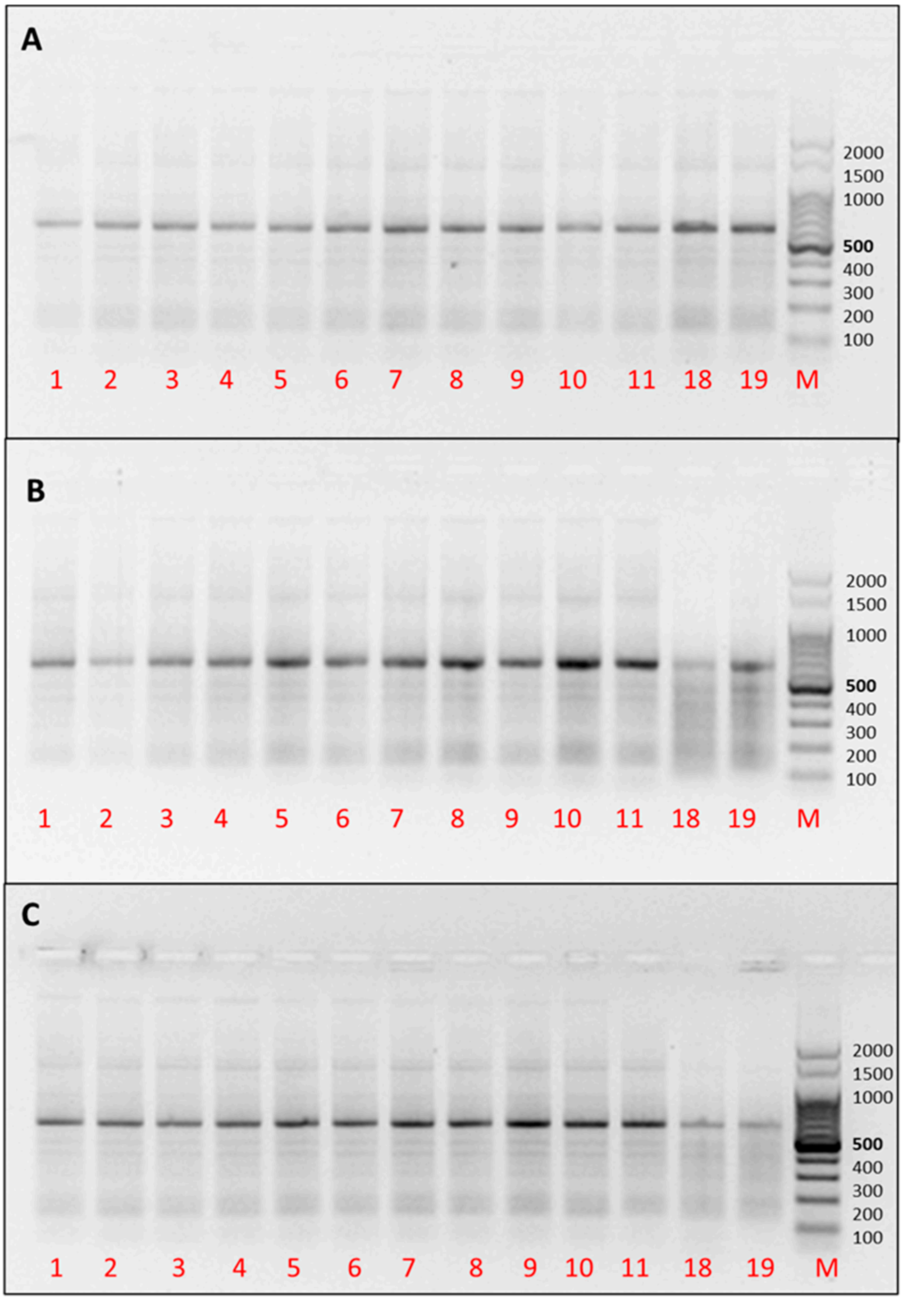

Supplementary Figure S1: Agarose gel electrophoresis of dsRNA conserved in A: water at 4°C; B: water at 32°C; C: sucrose at 32°C. The total volume of dsRNA was sampled in each lane. 1 to 19: days of conservation. M: molecular weight marker.

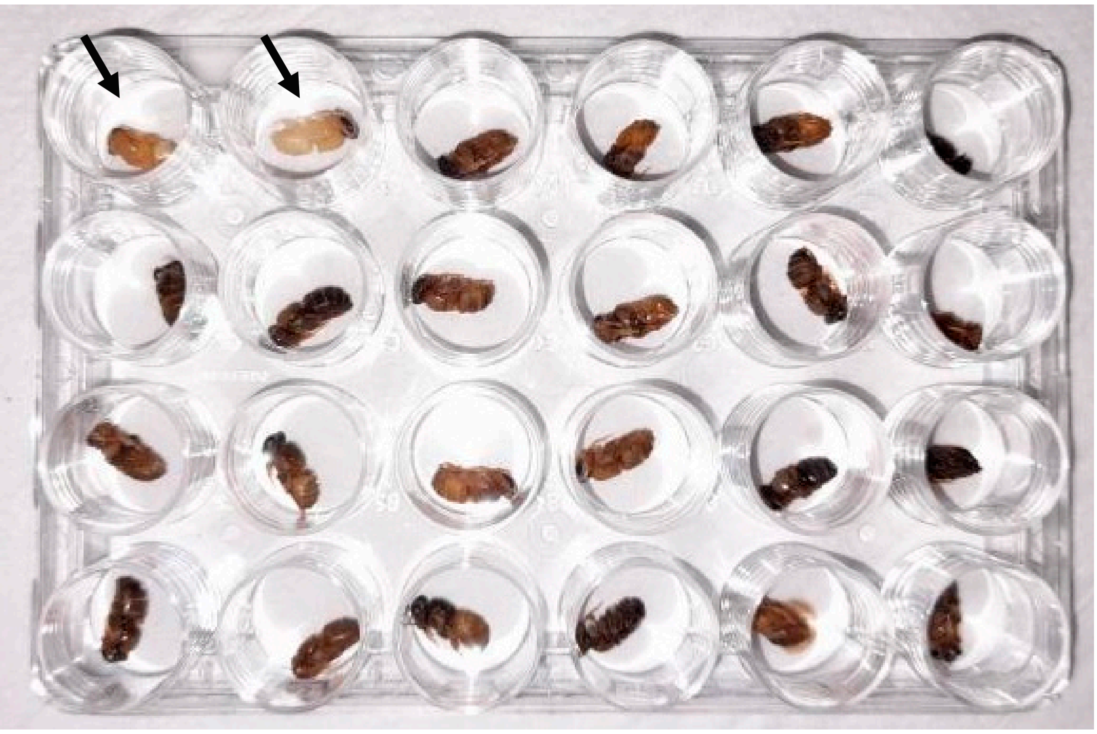

Supplementary Figure S2: *In vivo* virus amplification. Pupae of bees infected with virus on the third day post infection. Two pupae were injected with PBS, as a control of the procedure (marked with black arrows).

Pupae were collected from frames of emerging brood from to healthy colonies maintained at the research apiary of the Instituto de Genética Ewald A. Favret INTA-CONICET.

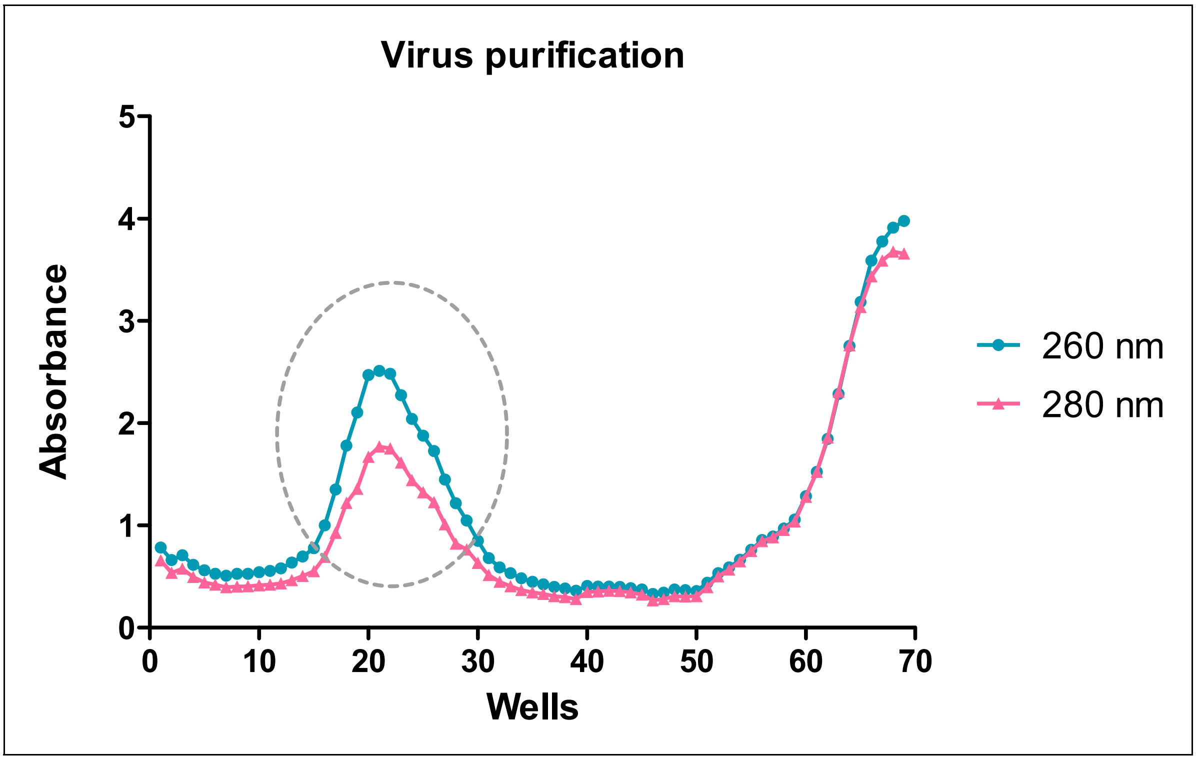

Supplementary Figure S3: Sucrose density gradient profile of ABPV particles produced in infected bee pupae. In each fraction protein and nucleic acid were assessed by measuring the absorbance at 280 and 260 nm, respectively.

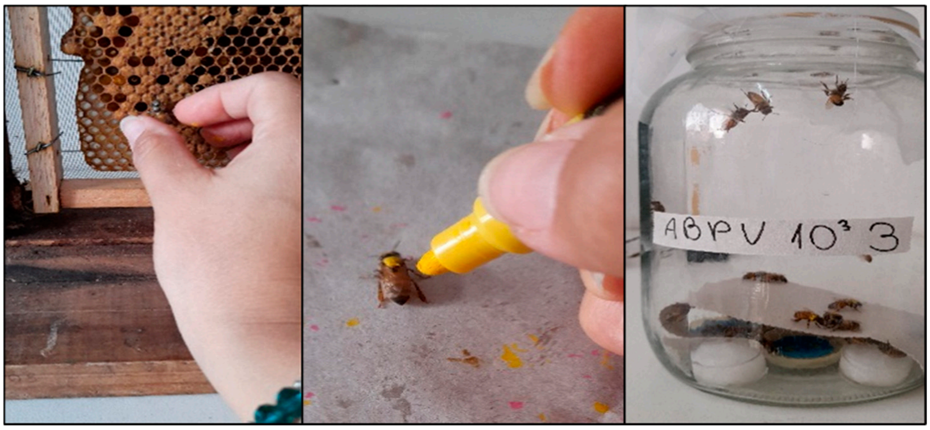

Supplementary Figure S4: Collection procedure and painting of recently emerged worker bees. Each jar (group) consisted of 50 adult bees: 30 bees were painted in yellow to record survival and 20 bees were painted in red to quantify viral loads.

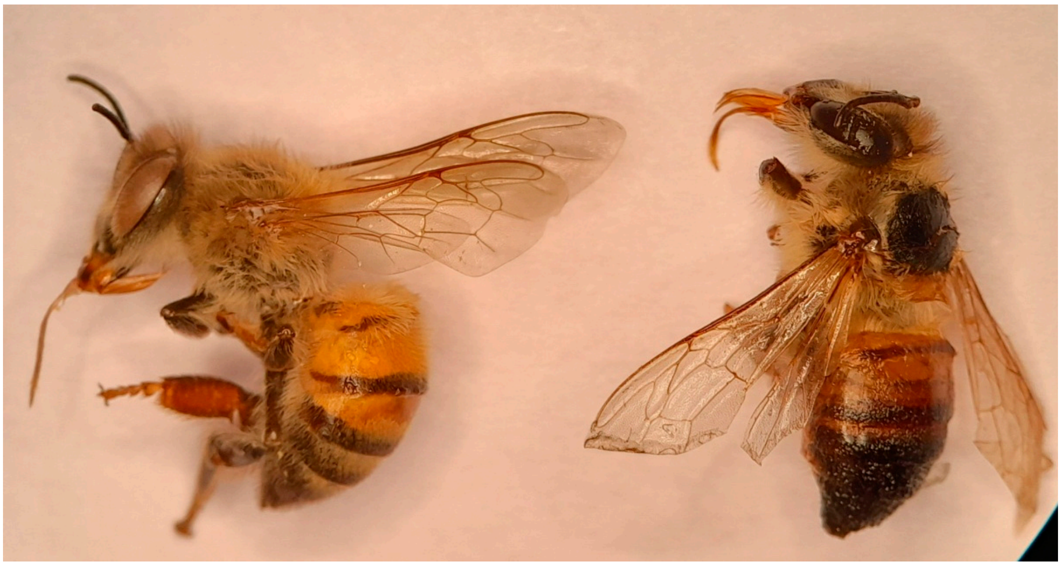

Supplementary Figure S5: Signs of ABPV infection. On the left a healthy bee and on the right an infected bee with hair loss on the thorax and abdomen, and gradual darkening.
